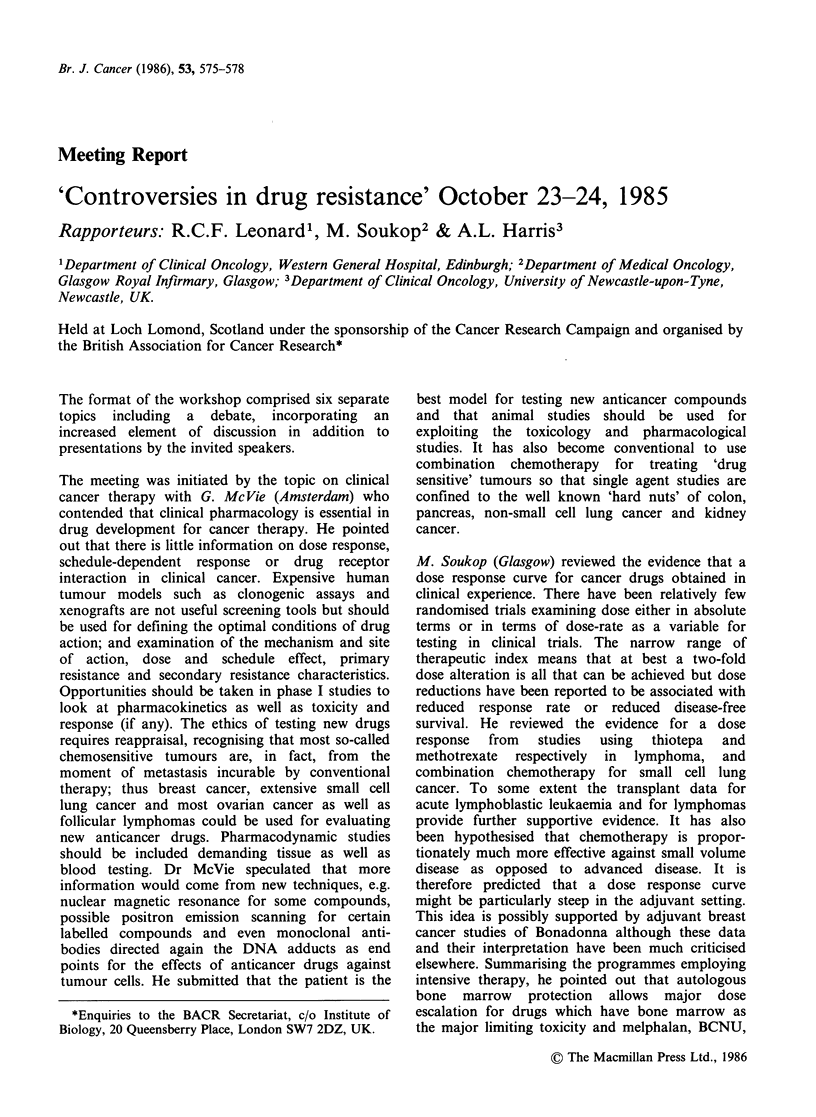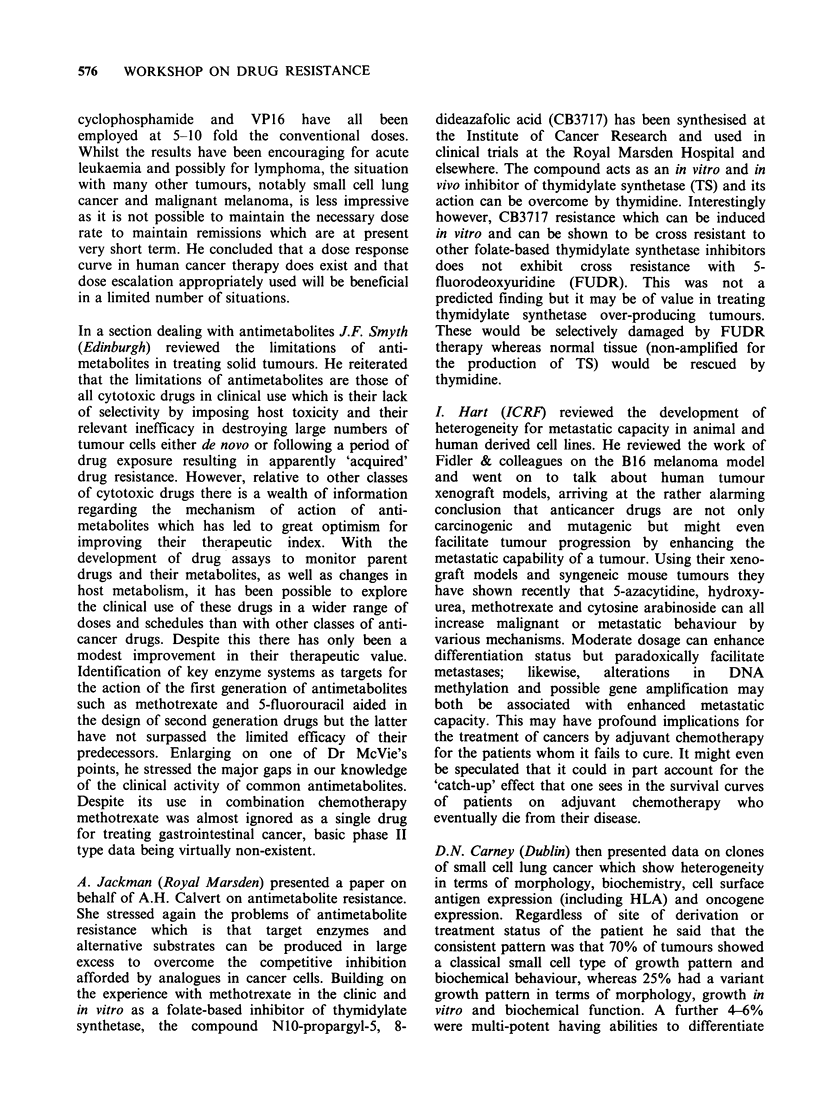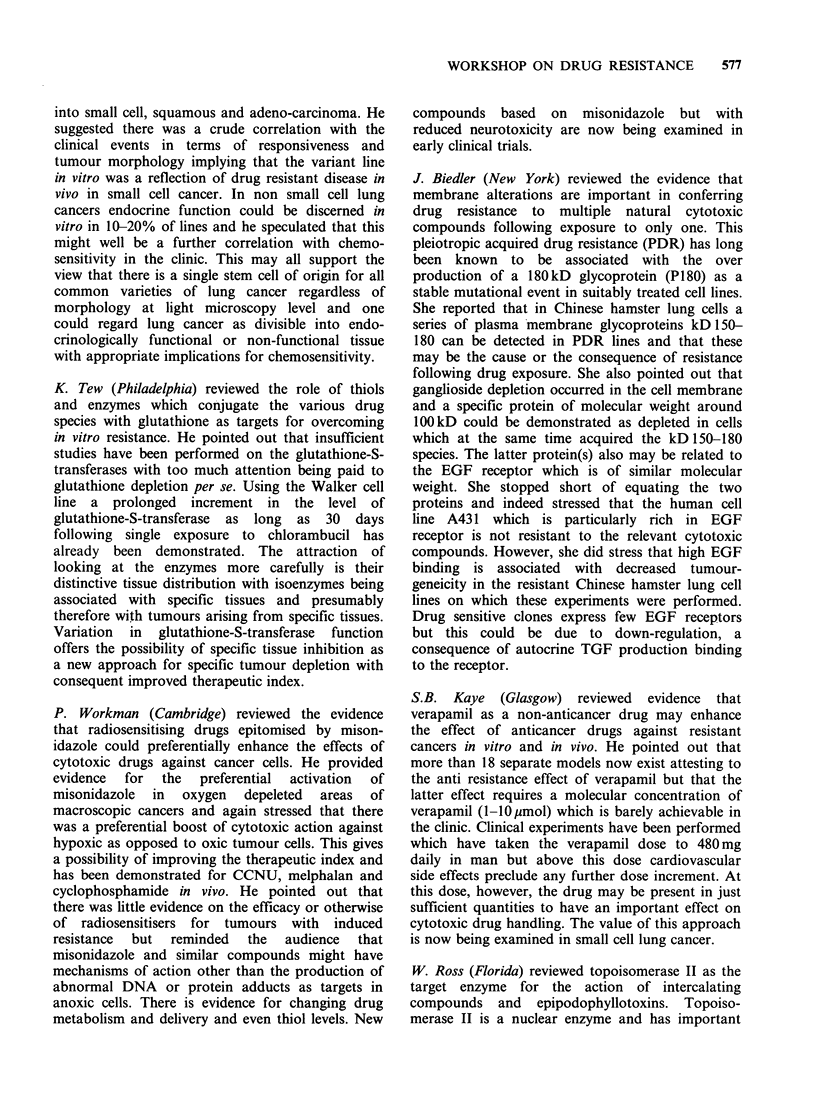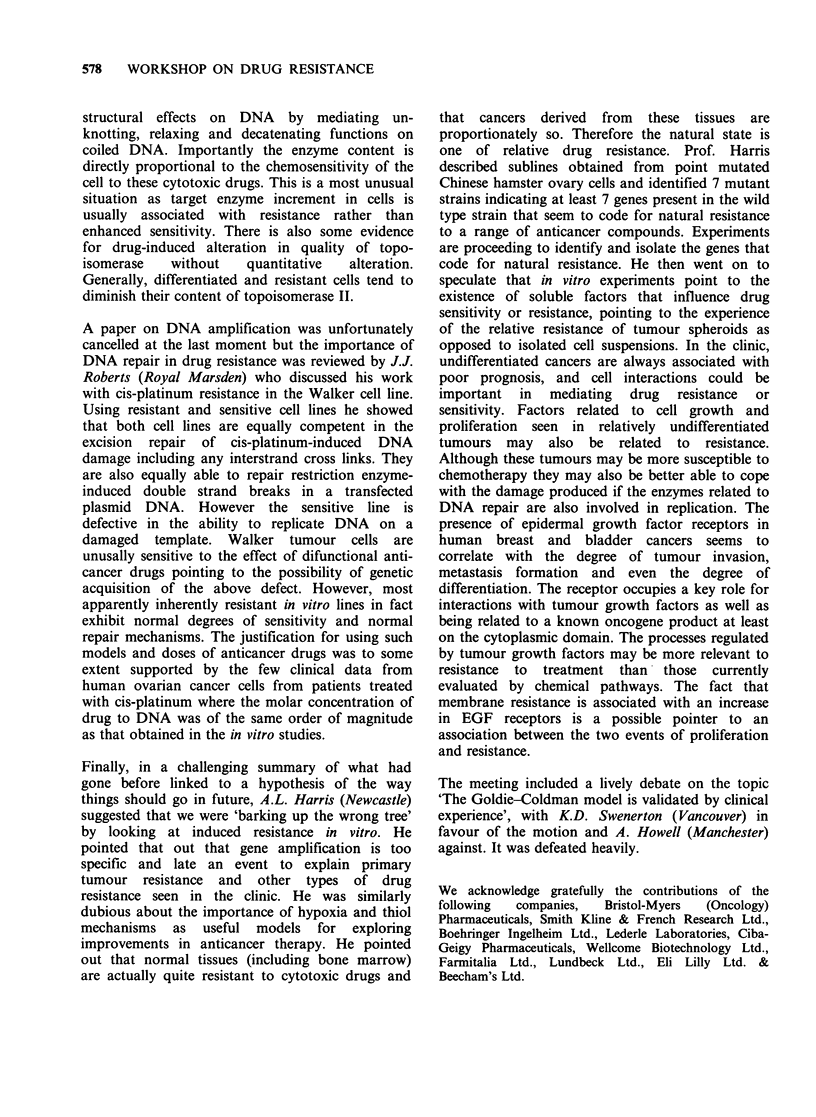# Report `Controversies in drug resistance' October 23-24, 1985

**Published:** 1986-04

**Authors:** 


Br. J. Cancer (1986), 53, 575–578

### Meeting Report

### 'Controversies in drug resistance' October 23–24, 1985

*Rapporteurs:* R.C.F. Leonard[1], M. Soukop[2] & A.L. Harris[3]

[1]*Department of Clinical Oncology, Western General Hospital, Edinburgh;* [2]*Department of Medical Oncology, Glasgow Royal Infirmary, Glasgow;* [3]*Department of Clinical Oncology, University of Newcastle-upon-Tyne, Newcastle, UK.*

Held at Loch Lomond, Scotland under the sponsorship of the Cancer Research Campaign and organised by the British Association for Cancer Research*

The format of the workshop comprised six separate topics including a debate, incorporating an increased element of discussion in addition to presentations by the invited speakers.

The meeting was initiated by the topic on clinical cancer therapy with *G. McVie* (*Amsterdam*) who contended that clinical pharmacology is essential in drug development for cancer therapy. He pointed out that there is little information on dose response, schedule-dependent response or drug receptor interaction in clinical cancer. Expensive human tumour models such as clonogenic assays and xenografts are not useful screening tools but should be used for defining the optimal conditions of drug action; and examination of the mechanism and site of action, dose and schedule effect, primary resistance and secondary resistance characteristics. Opportunities should be taken in phase I studies to look at pharmacokinetics as well as toxicity and response (if any). The ethics of testing new drugs requires reappraisal, recognising that most so-called chemosensitive tumours are, in fact, from the moment of metastasis incurable by conventional therapy; thus breast cancer, extensive small cell lung cancer and most ovarian cancer as well as follicular lymphomas could be used for evaluating new anticancer drugs. Pharmacodynamic studies should be included demanding tissue as well as blood testing. Dr McVie speculated that more information would come from new techniques, e.g. nuclear magnetic resonance for some compounds, possible positron emission scanning for certain labelled compounds and even monoclonal antibodies directed again the DNA adducts as end points for the effects of anticancer drugs against tumour cells. He submitted that the patient is the best model for testing new anticancer compounds and that animal studies should be used for exploiting the toxicology and pharmacological studies. It has also become conventional to use combination chemotherapy for treating 'drug sensitive' tumours so that single agent studies are confined to the well known 'hard nuts' of colon, pancreas, non-small cell lung cancer and kidney cancer.

*M. Soukop* (*Glasgow*) reviewed the evidence that a dose response curve for cancer drugs obtained in clinical experience. There have been relatively few randomised trials examining dose either in absolute terms or in terms of dose-rate as a variable for testing in clinical trials. The narrow range of therapeutic index means that at best a two-fold dose alteration is all that can be achieved but dose reductions have been reported to be associated with reduced response rate or reduced disease-free survival. He reviewed the evidence for a dose response from studies using thiotepa and methotrexate respectively in lymphoma, and combination chemotherapy for small cell lung cancer. To some extent the transplant data for acute lymphoblastic leukaemia and for lymphomas provide further supportive evidence. It has also been hypothesised that chemotherapy is proportionately much more effective against small volume disease as opposed to advanced disease. It is therefore predicted that a dose response curve might be particularly steep in the adjuvant setting. This idea is possibly supported by adjuvant breast cancer studies of Bonadonna although these data and their interpretation have been much criticised elsewhere. Summarising the programmes employing intensive therapy, he pointed out that autologous bone marrow protection allows major dose escalation for drugs which have bone marrow as the major limiting toxicity and melphalan, BCNU,

*Enquiries to the BACR Secretariat, c/o Institute of Biology, 20 Queensberry Place, London SW7 2DZ, UK.

© The Macmillan Press Ltd., 1986

cyclophosphamide and VP16 have all been employed at 5–10 fold the conventional doses. Whilst the results have been encouraging for acute leukaemia and possibly for lymphoma, the situation with many other tumours, notably small cell lung cancer and malignant melanoma, is less impressive as it is not possible to maintain the necessary dose rate to maintain remissions which are at present very short term. He concluded that a dose response curve in human cancer therapy does exist and that dose escalation appropriately used will be beneficial in a limited number of situations.

In a section dealing with antimetabolites *J.F. Smyth* (*Edinburgh*) reviewed the limitations of antimetabolites in treating solid tumours. He reiterated that the limitations of antimetabolites are those of all cytotoxic drugs in clinical use which is their lack of selectivity by imposing host toxicity and their relevant inefficacy in destroying large numbers of tumour cells either *de novo* or following a period of drug exposure resulting in apparently 'acquired' drug resistance. However, relative to other classes of cytotoxic drugs there is a wealth of information regarding the mechanism of action of antimetabolites which has led to great optimism for improving their therapeutic index. With the development of drug assays to monitor parent drugs and their metabolites, as well as changes in host metabolism, it has been possible to explore the clinical use of these drugs in a wider range of doses and schedules than with other classes of anticancer drugs. Despite this there has only been a modest improvement in their therapeutic value. Identification of key enzyme systems as targets for the action of the first generation of antimetabolites such as methotrexate and 5-fluorouracil aided in the design of second generation drugs but the latter have not surpassed the limited efficacy of their predecessors. Enlarging on one of Dr McVie's points, he stressed the major gaps in our knowledge of the clinical activity of common antimetabolites. Despite its use in combination chemotherapy methotrexate was almost ignored as a single drug for treating gastrointestinal cancer, basic phase II type data being virtually non-existent.

*A. Jackman* (*Royal Marsden*) presented a paper on behalf of A.H. Calvert on antimetabolite resistance. She stressed again the problems of antimetabolite resistance which is that target enzymes and alternative substrates can be produced in large excess to overcome the competitive inhibition afforded by analogues in cancer cells. Building on the experience with methotrexate in the clinic and *in vitro* as a folate-based inhibitor of thymidylate synthetase, the compound N10-propargyl-5, 8-dideazafolic acid (CB3717) has been synthesised at the Institute of Cancer Research and used in clinical trials at the Royal Marsden Hospital and elsewhere. The compound acts as an *in vitro* and *in vivo* inhibitor of thymidylate synthetase (TS) and its action can be overcome by thymidine. Interestingly however, CB3717 resistance which can be induced *in vitro* and can be shown to be cross resistant to other folate-based thymidylate synthetase inhibitors does not exhibit cross resistance with 5-fluorodeoxyuridine (FUDR). This was not a predicted finding but it may be of value in treating thymidylate synthetase over-producing tumours. These would be selectively damaged by FUDR therapy whereas normal tissue (non-amplified for the production of TS) would be rescued by thymidine.

*I. Hart* (*ICRF*) reviewed the development of heterogeneity for metastatic capacity in animal and human derived cell lines. He reviewed the work of Fidler & colleagues on the B16 melanoma model and went on to talk about human tumour xenograft models, arriving at the rather alarming conclusion that anticancer drugs are not only carcinogenic and mutagenic but might even facilitate tumour progression by enhancing the metastatic capability of a tumour. Using their xenograft models and syngeneic mouse tumours they have shown recently that 5-azacytidine, hydroxyurea, methotrexate and cytosine arabinoside can all increase malignant or metastatic behaviour by various mechanisms. Moderate dosage can enhance differentiation status but paradoxically facilitate metastases; likewise, alterations in DNA methylation and possible gene amplification may both be associated with enhanced metastatic capacity. This may have profound implications for the treatment of cancers by adjuvant chemotherapy for the patients whom it fails to cure. It might even be speculated that it could in part account for the 'catch-up' effect that one sees in the survival curves of patients on adjuvant chemotherapy who eventually die from their disease.

*D.N. Carney* (*Dublin*) then presented data on clones of small cell lung cancer which show heterogeneity in terms of morphology, biochemistry, cell surface antigen expression (including HLA) and oncogene expression. Regardless of site of derivation or treatment status of the patient he said that the consistent pattern was that 70% of tumours showed a classical small cell type of growth pattern and biochemical behaviour, whereas 25% had a variant growth pattern in terms of morphology, growth *in vitro* and biochemical function. A further 4–6% were multi-potent having abilities to differentiate into small cell, squamous and adeno-carcinoma. He suggested there was a crude correlation with the clinical events in terms of responsiveness and tumour morphology implying that the variant line *in vitro* was a reflection of drug resistant disease *in vivo* in small cell cancer. In non small cell lung cancers endocrine function could be discerned *in vitro* in 10–20% of lines and he speculated that this might well be a further correlation with chemosensitivity in the clinic. This may all support the view that there is a single stem cell of origin for all common varieties of lung cancer regardless of morphology at light microscopy level and one could regard lung cancer as divisible into endocrinologically functional or non-functional tissue with appropriate implications for chemosensitivity.

*K. Tew* (*Philadelphia*) reviewed the role of thiols and enzymes which conjugate the various drug species with glutathione as targets for overcoming *in vitro* resistance. He pointed out that insufficient studies have been performed on the glutathione-S-transferases with too much attention being paid to glutathione depletion *per se*. Using the Walker cell line a prolonged increment in the level of glutathione-S-transferase as long as 30 days following single exposure to chlorambucil has already been demonstrated. The attraction of looking at the enzymes more carefully is their distinctive tissue distribution with isoenzymes being associated with specific tissues and presumably therefore with tumours arising from specific tissues. Variation in glutathione-S-transferase function offers the possibility of specific tissue inhibition as a new approach for specific tumour depletion with consequent improved therapeutic index.

*P. Workman* (*Cambridge*) reviewed the evidence that radiosensitising drugs epitomised by misonidazole could preferentially enhance the effects of cytotoxic drugs against cancer cells. He provided evidence for the preferential activation of misonidazole in oxygen depeleted areas of macroscopic cancers and again stressed that there was a preferential boost of cytotoxic action against hypoxic as opposed to oxic tumour cells. This gives a possibility of improving the therapeutic index and has been demonstrated for CCNU, melphalan and cyclophosphamide *in vivo*. He pointed out that there was little evidence on the efficacy or otherwise of radiosensitisers for tumours with induced resistance but reminded the audience that misonidazole and similar compounds might have mechanisms of action other than the production of abnormal DNA or protein adducts as targets in anoxic cells. There is evidence for changing drug metabolism and delivery and even thiol levels. New compounds based on misonidazole but with reduced neurotoxicity are now being examined in early clinical trials.

*J. Biedler* (*New York*) reviewed the evidence that membrane alterations are important in conferring drug resistance to multiple natural cytotoxic compounds following exposure to only one. This pleiotropic acquired drug resistance (PDR) has long been known to be associated with the over production of a 180 kD glycoprotein (P180) as a stable mutational event in suitably treated cell lines. She reported that in Chinese hamster lung cells a series of plasma membrane glycoproteins kD 150–180 can be detected in PDR lines and that these may be the cause or the consequence of resistance following drug exposure. She also pointed out that ganglioside depletion occurred in the cell membrane and a specific protein of molecular weight around 100 kD could be demonstrated as depleted in cells which at the same time acquired the kD 150–180 species. The latter protein(s) also may be related to the EGF receptor which is of similar molecular weight. She stopped short of equating the two proteins and indeed stressed that the human cell line A431 which is particularly rich in EGF receptor is not resistant to the relevant cytotoxic compounds. However, she did stress that high EGF binding is associated with decreased tumourgeneicity in the resistant Chinese hamster lung cell lines on which these experiments were performed. Drug sensitive clones express few EGF receptors but this could be due to down-regulation, a consequence of autocrine TGF production binding to the receptor.

*S.B. Kaye* (*Glasgow*) reviewed evidence that verapamil as a non-anticancer drug may enhance the effect of anticancer drugs against resistant cancers *in vitro* and *in vivo*. He pointed out that more than 18 separate models now exist attesting to the anti resistance effect of verapamil but that the latter effect requires a molecular concentration of verapamil (1–10 $\mu$mol) which is barely achievable in the clinic. Clinical experiments have been performed which have taken the verapamil dose to 480 mg daily in man but above this dose cardiovascular side effects preclude any further dose increment. At this dose, however, the drug may be present in just sufficient quantities to have an important effect on cytotoxic drug handling. The value of this approach is now being examined in small cell lung cancer.

*W. Ross* (*Florida*) reviewed topoisomerase II as the target enzyme for the action of intercalating compounds and epipodophyllotoxins. Topoisomerase II is a nuclear enzyme and has important structural effects on DNA by mediating unknotting, relaxing and decatenating functions on coiled DNA. Importantly the enzyme content is directly proportional to the chemosensitivity of the cell to these cytotoxic drugs. This is a most unusual situation as target enzyme increment in cells is usually associated with resistance rather than enhanced sensitivity. There is also some evidence for drug-induced alteration in quality of topoisomerase without quantitative alteration. Generally, differentiated and resistant cells tend to diminish their content of topoisomerase II.

A paper on DNA amplification was unfortunately cancelled at the last moment but the importance of DNA repair in drug resistance was reviewed by *J.J. Roberts* (*Royal Marsden*) who discussed his work with cis-platinum resistance in the Walker cell line. Using resistant and sensitive cell lines he showed that both cell lines are equally competent in the excision repair of cis-platinum-induced DNA damage including any interstrand cross links. They are also equally able to repair restriction enzyme-induced double strand breaks in a transfected plasmid DNA. However the sensitive line is defective in the ability to replicate DNA on a damaged template. Walker tumour cells are unusally sensitive to the effect of difunctional anticancer drugs pointing to the possibility of genetic acquisition of the above defect. However, most apparently inherently resistant *in vitro* lines in fact exhibit normal degrees of sensitivity and normal repair mechanisms. The justification for using such models and doses of anticancer drugs was to some extent supported by the few clinical data from human ovarian cancer cells from patients treated with cis-platinum where the molar concentration of drug to DNA was of the same order of magnitude as that obtained in the *in vitro* studies.

Finally, in a challenging summary of what had gone before linked to a hypothesis of the way things should go in future, *A.L. Harris* (*Newcastle*) suggested that we were 'barking up the wrong tree' by looking at induced resistance *in vitro*. He pointed that out that gene amplification is too specific and late an event to explain primary tumour resistance and other types of drug resistance seen in the clinic. He was similarly dubious about the importance of hypoxia and thiol mechanisms as useful models for exploring improvements in anticancer therapy. He pointed out that normal tissues (including bone marrow) are actually quite resistant to cytotoxic drugs and that cancers derived from these tissues are proportionately so. Therefore the natural state is one of relative drug resistance. Prof. Harris described sublines obtained from point mutated Chinese hamster ovary cells and identified 7 mutant strains indicating at least 7 genes present in the wild type strain that seem to code for natural resistance to a range of anticancer compounds. Experiments are proceeding to identify and isolate the genes that code for natural resistance. He then went on to speculate that *in vitro* experiments point to the existence of soluble factors that influence drug sensitivity or resistance, pointing to the experience of the relative resistance of tumour spheroids as opposed to isolated cell suspensions. In the clinic, undifferentiated cancers are always associated with poor prognosis, and cell interactions could be important in mediating drug resistance or sensitivity. Factors related to cell growth and proliferation seen in relatively undifferentiated tumours may also be related to resistance. Although these tumours may be more susceptible to chemotherapy they may also be better able to cope with the damage produced if the enzymes related to DNA repair are also involved in replication. The presence of epidermal growth factor receptors in human breast and bladder cancers seems to correlate with the degree of tumour invasion, metastasis formation and even the degree of differentiation. The receptor occupies a key role for interactions with tumour growth factors as well as being related to a known oncogene product at least on the cytoplasmic domain. The processes regulated by tumour growth factors may be more relevant to resistance to treatment than those currently evaluated by chemical pathways. The fact that membrane resistance is associated with an increase in EGF receptors is a possible pointer to an association between the two events of proliferation and resistance.

The meeting included a lively debate on the topic 'The Goldie–Coldman model is validated by clinical experience', with *K.D. Swenerton* (*Vancouver*) in favour of the motion and *A. Howell* (*Manchester*) against. It was defeated heavily.

We acknowledge gratefully the contributions of the following companies, Bristol-Myers (Oncology) Pharmaceuticals, Smith Kline & French Research Ltd., Boehringer Ingelheim Ltd., Lederle Laboratories, Ciba-Geigy Pharmaceuticals, Wellcome Biotechnology Ltd., Farmitalia Ltd., Lundbeck Ltd., Eli Lilly Ltd. & Beecham's Ltd.